# Metallic Component Preserving Algorithm Based on the Cerebral Computed Tomography Angiography in Aneurysm Surgery

**DOI:** 10.3390/diagnostics12020338

**Published:** 2022-01-28

**Authors:** Jina Shim, Su Hwan Lee, Youngjin Lee, Kyu Bom Kim, Kyuseok Kim

**Affiliations:** 1 Department of Radiology and Research, Institute of Radiological Science, Severance Hospital, Yonsei University College of Medicine, Yonsei-ro, Seodaemun-gu, Seoul 03722, Korea; EOEORNFL@yuhs.ac; 2 Division of Pulmonary and Critical Care Medicine, Department of Internal Medicine, Severance Hospital, Yonsei University College of Medicine, Yonsei-ro, Seodaemun-gu, Seoul 03722, Korea; HIHOGOGO@yuhs.ac; 3 Department of Radiological Science, Gachon University, 191, Hambakmoe-ro, Yeonsu-gu, Incheon 21936, Korea; yj20@gachon.ac.kr; 4 Department of Integrative Medicine, Major in Digital Healthcare, Yonsei University College of Medicine, Unju-ro, Gangman-gu, Seoul 06229, Korea

**Keywords:** cerebral CT angiography (CTA), bone subtraction, aneurysm, clip and coil, performance evaluation

## Abstract

The purpose of this study was to investigate the viability of the proposed method in preventing the loss of metallic components including the clip and coil in cerebral computed tomography angiography (CTA). Forty patients undergoing surgery for aneurysms carried metallic materials. The proposed method is based on conventional bone subtraction CTA (BS-CTA) system. Briefly, the position of metal components was determined using the threshold value and a region of interest (ROI). An appropriate threshold was used to separate the background from the target materials based on the Otsu method. A three-dimensional (3D) rendering was performed from the proposed BS-CTA data carrying the extracted target information. The accuracy of clip and coil region measured using the dice similarity coefficient (DSC) and bidirectional Hausdorff distance (HD) is reported. The metallic components of the proposed BS-CTA were significantly visualized in various patient cases. Quantitative evaluation using the proposed method is based on the mean DSC of 0.93 with a standard deviation (SD) of ±0.05 (e.g., maximum value = 0.99, minimum value = 0.75, 95% confidence interval (CI) = 0.91 to 0.95, and all *p* < 0.05). The mean HD was 1.50 voxels with an SD of ± 0.58 (e.g., maximum value = 5.95, minimum value = 0.12, 95% CI = 1.10 to 1.90, and all *p* < 0.05). The proposed method demonstrates effective segmentation of the metallic component and application to the existing conventional BS-CTA system.

## 1. Introduction

Early detection of cerebral aneurysms is most important in preventing subarachnoid hemorrhage (SAH). Computed tomography angiography (CTA) is a well-established technique used to detect this disease accurately and cost-effectively, compared with conventional digital subtraction angiography (DSA) and magnetic resonance angiography (MRA) in various clinical imaging applications [1,2]. Invasive three-dimensional (3D) DSA is the gold standard for detection of cerebral aneurysms. However, it can lead to permanent neurologic deficits in 0.12% of all patients [3] and may increase the risk of rebleeding, based on 5% to 10% false-negative results [4,5]. MRA is a non-invasive method without exposure to ionizing radiation and does not entail the use of contrast agents except in extraordinary cases. Nevertheless, it is susceptible to motion artefacts due to the long scan times required and higher scan costs compared with CTA [6,7].

**Figure 1:**
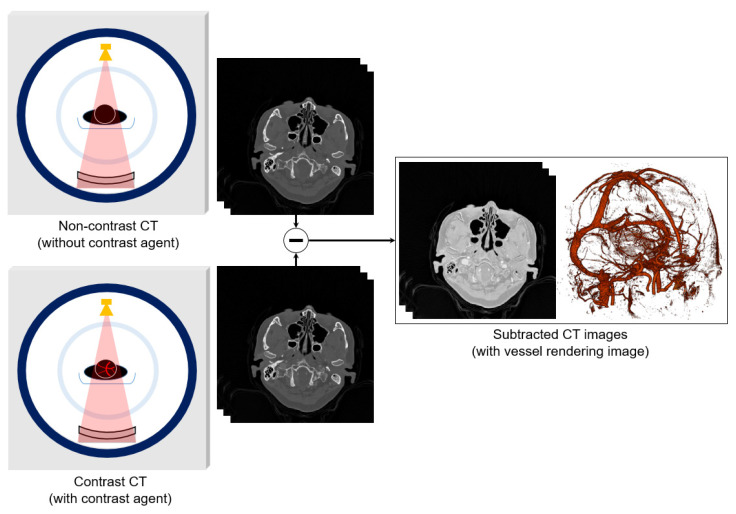
Acquisition of computed tomography angiography (CTA) images by directly subtracting non-contrast CT images without a contrast agent from contrast CT images. Vessel rendering showing segmentation of the subtracted CT images based on the Hounsfield unit (HU) values.

Conventionally, cerebral CTA can be classified into two types: (1) non-subtraction CTA (NS-CTA) and (2) bone subtraction CTA (BS-CTA) [3,8]. NS-CTA is performed using the multidetector-row CT when injecting the contrast agent, whereas BS-CTA requires twice CT scan with and without the injection of contrast medium under the same conditions. It is accurate in detecting cerebral aneurysms and in guiding rapid treatment decisions compared with NS-CTA [9]. The concept of BS-CTA technique is illustrated in Figure 1. The cerebral vessel can be segmented by directly subtracting the contrast-enhanced CT from noncontrast CT. However, the BS-CTA technique is inherently limited by motion artefacts and increasing radiation dose due to double exposure. The movement of the patient induces a physical error in the acquired image, and results in incomplete removal of bones, including the cervical bones, mandible, and hyoid. Methods of image registration (e.g., rigid, slab-based rigid, and partial rigid) yield outstanding results for accurate segmentation of vessels [10]. In addition, a variety of methods are being studied to improve the quality of low-dose CTA images to resolve the radiation dose challenges [11].

As a novel approach, dual-energy-based BS-CTA (DE-BS-CTA) is a well-established theoretical technique and is widely used in cerebral CTA [12,13]. This method is based on the two-dimensional (2D) projections of two different levels of X-ray energy (e.g., 80 kV_p_ and 140 kV_p_, or 100 kV_p_ and 140 kV_p_) to distinguish the types of materials based on a difference in attenuation coefficient according to the materials in question [14]. However, this approach inherently requires exposure to increased patient radiation doses, increased execution time, and motion artefacts due to the dual scan needed to project different values of kV_p_. A variety of approaches have been used to overcome these problems, including dual X-ray sources, rapid switch of X-ray tube method, a dual-layer detector, and a photon-counting detector [15]. These techniques can enhance the image quality compared with conventional DE-BS-CTA by minimizing the geometrical misregistration between both projections of low-kV_p_ and high-kV_p_ due to the single rotation used for the examination. Especially, the photon counting detector generates energy-based spectral CT images because the large electrical pulse generated by the semiconductor diode from incident X-rays is proportional to the energy of photon. It improves the performance of material decomposition and excludes the electronic noise effectively [16].

**Figure 2:**
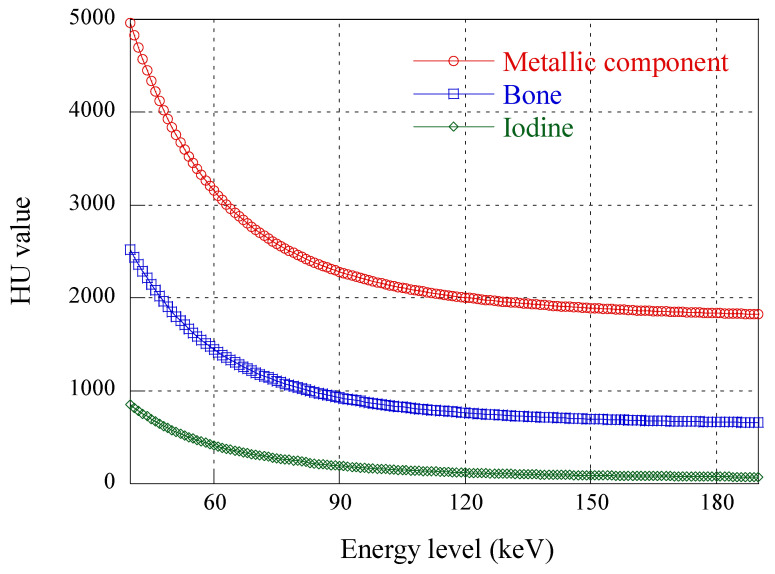
HU values plotted with metallic component, bone, and iodine in the range of 50 keV to 190 keV.

The BS-CTA and DE-BS-CTA are limited by the possible loss of clip or coil information. Clipping or coiling is a method used to treat aneurysms, and occlusion of aneurysms can minimize the risk of complications such as hemorrhage or aneurysm growth while preserving parent vessels. These mainly consist of alloys such as titanium and cobalt [17], which are eliminated in the post-processing of BS-CTA and DE-BS-CTA [18]. Since the clip image information is conveyed not only by the contrast CT but also non-contrast CT in BS-CTA, the clip along with the bone can be removed during the subtraction. DE-BS-CTA can also inherently eliminate the clip or coil. Figure 2 shows the Hounsfield units (HU) of the contrast medium, bone, and clip or coil according to the incident X-ray energy [19]. In general, the DE-BS-CTA method presents a basic function for material decomposition in advance to distinguish bones from contrast medium. In this case, when the HU value of the metallic clip or coil is equal to or higher than that of bone, it may be deemed a bony material and removed together [20].

The aim of this study was to assess the role of metallic clip or coil preservation in conventional BS-CTA. It is possible to obtain the vessel rendering image with minimal loss of information by selecting an area containing the metallic components. We implemented the proposed method and performed a clinical experiment to demonstrate its usefulness.

## 2. Materials and Methods

### 2.1. Proposed Framework for Metallic Clip or Coil Preservation in BS-CTA Image

**Figure 3:**
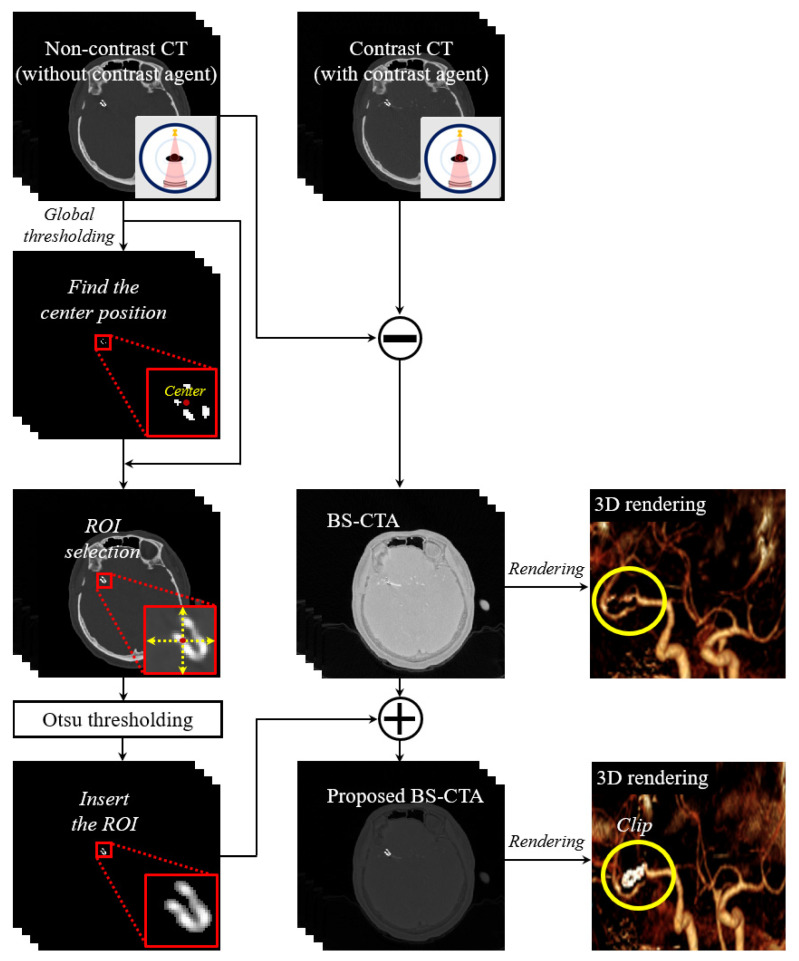
A simplified flowchart of proposed bone subtraction CTA (BS-CTA) method preserving clip and coil components.

Figure 3 shows the proposed framework to preserve the metallic clip or coil component in conventional BS-CTA images. In brief, high-intensity information extracted from non-contrast CT images above the global threshold value should be adequate to separate the bones from the metallic material, and the threshold value was set at 3500 HU via trial and error. The centers of the extracted components were calculated and the central coordinate (*CC*) was defined as follows:

(1) $$CC_{row}=\frac{\left(R_{max}-R_{min}\right)}{2},\;CC_{col}=\frac{\left(C_{max}-C_{min}\right)}{2},\;and\;\left(R\;and\;C\in \Omega \right),$$

where $CC_{row}$ and $CC_{col}$ denote the center positions of row (*R*) and column (*C*), respectively, among the extracted components, and Ω is the entire region in the slice image. The region of interest (ROI) is selected in the non-contrast CT slice and the ROI size is empirically set to the $2\alpha (R_{max}-CC_{row})\times 2\alpha (C_{max}-CC_{row})$. Here, α represents the control parameter to ensure the clip or coil component in ROI, and we used an α value of 1.5.

To specify the metal information alone, Otsu’s method [21] was used in the ROI to generate the binary image based on the appropriate threshold value in the histogram as follows:

(2) $$\sigma_{BC}^{2}=\frac{{\left(m_{G}P-m\right)}^{2}}{P\left(1-P\right)},\;m\left(k\right)=\overset{k}{\underset{i=0}{\sum }}iP_{i},\;\left(0<k\leq \Omega \right),$$

where $\sigma_{BC}^{2}$ is the between-class variance, *P* denotes the probability that the intensity of a pixel belongs to the class, and $m_{G}$ represents the average intensity of the image. Here, m indicates the average value, which ranges between 0 and *k* at all levels of intensity, Ω. The larger the difference in the average intensity between *p* values and vice versa, the greater the $\sigma_{BC}^{2}$. Thus, *k* is deduced from the between-class variance in the case of maximum value due to increased separation between classes. It was performed using the *graythresh* (∙) and *imbinarize* (∙) functions in MATLAB^TM^ (Mathworks, Natick, MA, USA, R2020a). This approach for extracting the ROI is not limited to Otsu’s method, and an example is presented. Finally, the ROI information was incorporated into BS-CTA image, which was obtained by directly subtracting the non-contrast CT images from the contrast CT images. We implemented the proposed algorithm with a normal workstation (OS: Windows 10 (Microsoft, Redmond, WA, USA), CPU: Intel Xeon (Intel, Santa Clara, CA, USA), 2.13 GHz, RAM: 64 GB (Samsung, Suwan-si, South Korea)), and the processing time takes about 0.9 s per reconstruction slice using the only CPU processor.

### 2.2. Patient Cohort

This retrospective study was approved by the institutional review board (4-2020-1364 and 26 January 2021) of Severance Hospital, and the requirement for informed consent was waived due to the retrospective design of the study. This study evaluated adult patients with post-cerebral aneurysm clipping and coiling. Forty patients (34 females and 6 males, mean age 59 years, from 26 to 74 years) underwent cerebral CTA, including non-contrast CT, between 2019 and 2021. The distribution of the clipping and coiling cases was as follows: eight anterior cerebral artery (ACA), 23 middle cerebral arteries (MCA), 15 internal carotid arteries (ICA), one basilar artery (BA) and one posterior cerebral artery (PCA). Thirty-two patients were treated with a single clip and seven patients with multiple clips, while two patients were treated for coiling and two others for multiple clipping and coiling.

### 2.3. CT Acquisition and Post Processing

Cerebral non-contrast CT and contrast CTA slices were acquired with a Siemens SOMATOM FORCE CT scanner (Siemens Healthcare AG, Erlangen, Germany) and a similar scan range were adopted from cranial base to calvaria. The CT scan was performed using the following parameters: an X-ray tube (100 kV_p_, single source), beam collimation (0.6 mm), matrix size (512 × 512 × 320), slice thickness (0.75 and 1 mm), rotation time (0.5 s), and reconstruction kernel (Hv 40 and Hv 49) using iterative reconstruction (ADMIRE 1). Here, the cerebral CTA was implemented via intravenous injection of 100 mL at a rate of 4.0 mL per second using a power injector of iodinated contrast media (Iopamiro 370, Bracco, Milan, Italy), followed by a bolus injection of 40 mL of saline chaser. The detailed CT scan specifications are listed in Table 1.

The CT dose index (CTDI) volume and dose length product (DLP) were recorded. The mean CTDI and DLP values of 40 patients were 29.12 (e.g., maximum value = 29.20 and minimum value = 26.14) and 538.25 (e.g., maximum value = 591.80 and minimum value = 451.00), respectively.

### 2.4. Image Analysis

To evaluate the accuracy of segmentation results of the coiling and clipping regions, the the Dice similarity coefficient (*DSC*) [22,23] and bidirectional Hausdorff distance (HD) [24,25] were used. DICE is the most representative method, which is based on the overlap between the reference image (*X*) and the predicted image (*Y*). It is defined by the following equation:

(3) $$DSC\left(X,Y\right)=\frac{2\left|X⋂Y\right|}{\left|X\right|+\left|Y\right|},$$

where |∙| denotes cardinality, and the value of DICE is higher when the predicted image is similar to the reference image. Here, DICE factor is only determined by the overlapping area (e.g., false positives and false negatives) and it is difficult to reflect voxels without overlapping sections. In order to increase the reliability of the results, we measured the one-sided Hausdorff distance, *hd*, which is the spatial distance-based evaluation factor expressed as follows:

(4) $$hd\left(X,Y\right)=\underset{x\in X}{\max}\underset{y\in Y}{\min}\|x-y\|,\;hd\left(Y,X\right)=\underset{y\in Y}{\max}\underset{x\in X}{\min}\|x-y\|,$$

where ‖∙‖ denotes any norm, we used the Euclidean distance. Here, hd(X,Y)≠hd(Y,X), in most cases, and HD represents the maximum value in both directed *hd* (∙) values. The value of HD can be interpreted as the mean error between the *X* and *Y* boundaries.

### 2.5. Statistical Analysis

The quantitative variables of DSC and HD for each CTA slice were reported as the mean ± standard deviation (SD), median, minimum and maximum values, and 95% confidence intervals (CI). Data analysis was conducted with SPSS software (version 20.0, SPSS Inc., Chicago, IL, USA), and a *p*-value of ≤0.05 was considered to indicate statistical significance.

## 3. Results and Discussion

**Figure 4:**
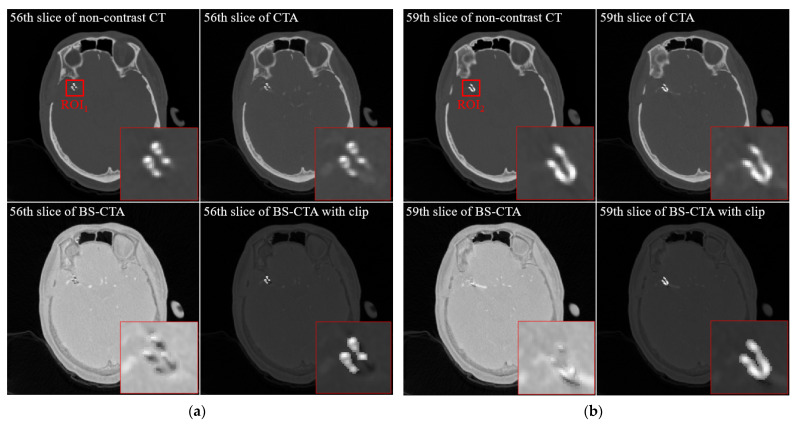
Examples of the non-contrast CT (**left upper**), the CTA (**right upper**), the BS-CTA (**left bottom**)**,** and the proposed BS-CTA (**right bottom**); (**a**) 56th slice and (**b**) 59th slice.

**Figure 5:**
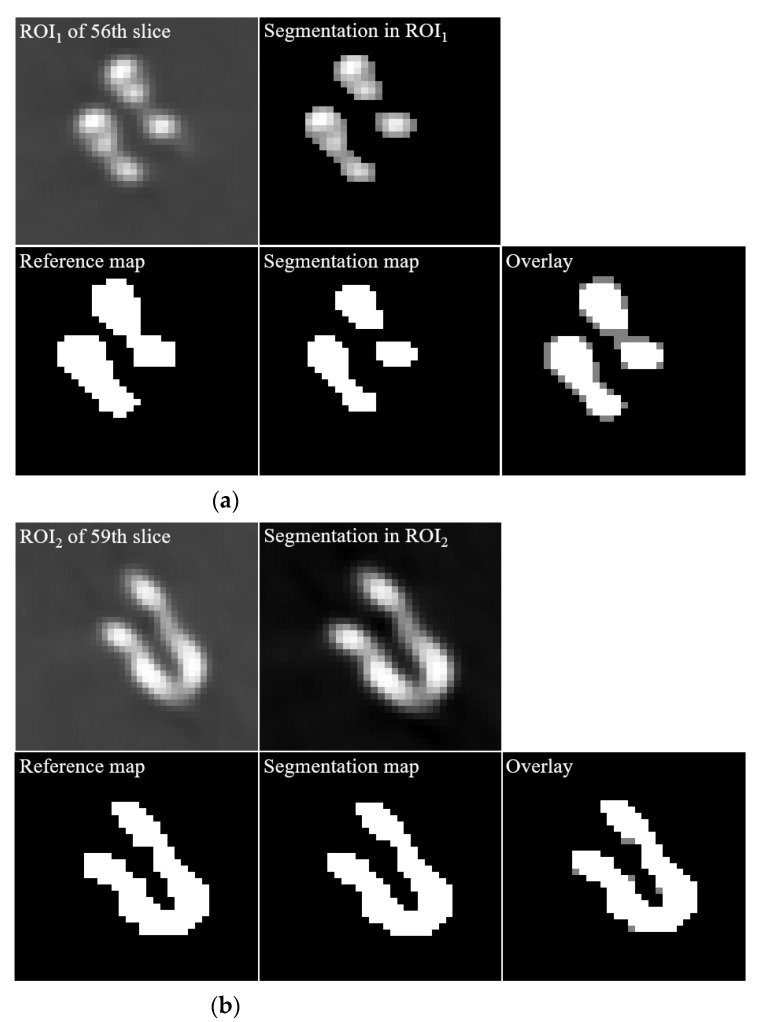
The enlarged images of (**a**) a ROI_1_ of 56th slice (**upper left**) and image segmentation in ROI_1_ (**upper middle**) represent the complete results of clip segmentation, a reference map (**bottom left**), a segmentation map (**bottom middle**) using the proposed method, and an image overlay (bottom right) of both reference and segmentation maps. (**b**) These are also the resultant images in ROI_2_ of 59th slice as (**a**).

**Figure 6:**
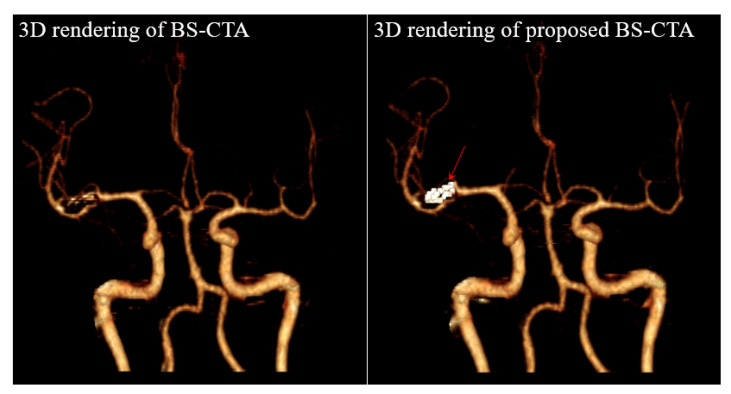
Results of 3D rendering of conventional BS-CTA (**left**) and proposed BS-CTA (**right**).

Figure 4 shows the results of a slice of non-contrast CT (upper left), a similar slice of CTA (upper right), a similar slice of BS-CTA (bottom left), and a proposed slice of BS-CTA with clip (bottom right): (a) 56th slice and (b) 59th slice. In general, most of them are eliminated in BS-CTA except for vascular information with contrast agents. The remaining residual substance of the clip was attributed to the difference in intensity due to external factors, including the contrast medium injected, image reconstruction and registration. The proposed results show the preservation of metallic components. Figure 5 presents sample segmentation images using the proposed method: (a) a ROI_1_ of 56th slice (upper left) is enlarged in Figure 4a and a segmentation image in ROI_1_ (upper middle) shows the complete results of clip segmentation. To evaluate the accuracy of the proposed method, an image overlay (bottom right) shows the qualitative concordance rate between a reference map (bottom left) and a segmentation map (bottom middle). Here, these are binary maps of clip region and reference maps were examined by three radiographers, including J. Shim with more than ten years of work experience, Y. Lee, and K. Kim. The image (b) represents an ROI_2_ of the 59th slice and is in the same order as (a). The results of Figure 4 and Figure 5 indicate the effectiveness of the proposed method when the single clip was inserted into a non-contrast CT slice. In addition, the proposed method was implemented in the non-contrast CT slice following the extraction of the brain vessels with contrast medium. It can be difficult to delimit the target area in the threshold techniques when there is little difference in HU value between the metallic component and blood vessel treated with the contrast agent. Therefore, the proposed method was designed by extracting the metallic components from a non-contrast CT slice. In addition, the proposed method did not utilize the post-image processing blending the extracted component and the BS-CTA slice. The aim of the BS-CTA technique was to visualize the three-dimensional (3D) vessel data. Thus, the BS-CTA slice containing metallic components was hardly used when evaluating cerebral blood vessels for abnormality. Therefore, the extracted information was simply incorporated into the BS-CTA slice in order to prevent distortion that may occur during further image processing. The proposed method yields high-performance metrics. The mean DSC was 0.92 with an SD of ±0.05 (e.g., maximum value = 0.98, minimum value = 0.84, 95% CI = 0.88 to 0.95, and *p* = 0.0017) and the mean HD was 2.58 voxels with an SD of ± 1.42 (e.g., maximum value = 5.07, minimum value = 1.02, 95% CI = 0.81 to 3.00, *p* = 0.0052). Here, the values of mean DSC and mean HD were calculated from the 55th and 63rd slices containing the metallic component. The results are summarized in Table 2. Figure 6 shows the results of 3D rendering based on BS-CTA data (left) and the proposed BS-CTA data with the clip component (right). It suggests that the proposed method appropriately extracted the effective region of the clip. In particular, it is possible to increase the diagnostic value of the metallic component, especially in patients undergoing surgery for aneurysms.

**Figure 7:**
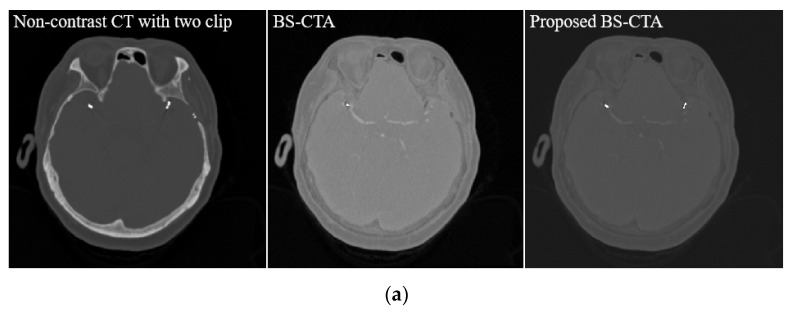

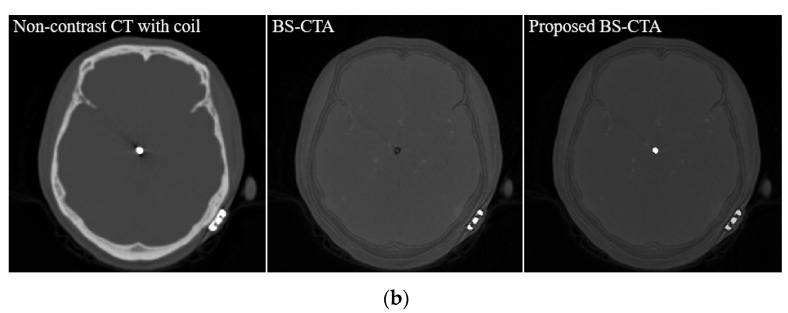
Results of non-contrast CT (**left**), BS-CTA (**middle**), and the proposed BS-CTA (**right**); (**a**) the two-clip case and (**b**) the coil case.

**Figure 8:**
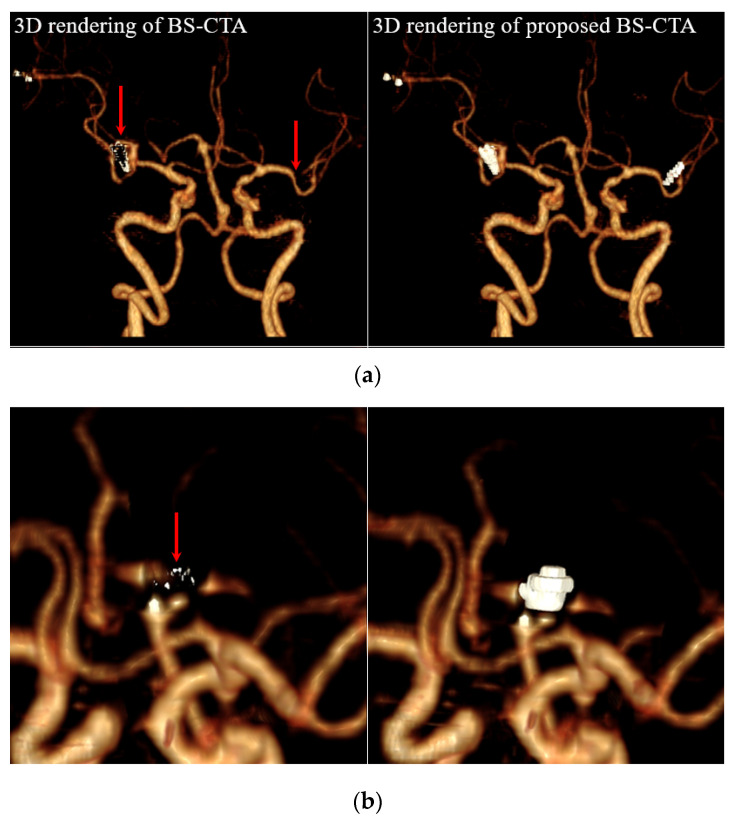
Results of 3D rendering based on BS-CTA and proposed BS-CTA from Figure 7. (**a**) Insertion of the two clips and (**b**) the coil components.

Figure 7 illustrates non-contrast CT (left), BS-CTA (middle), and the proposed BS-CTA (right): (a) the two-clip case and (b) the coil case. A significant loss of metallic material occurred in the proposed BS-CTA compared with that of conventional BS-CTA. In the case of a slice with an inserted coil, the overwhelming results are attributed due to the strong distribution intensity in the narrow region. In addition, Figure 7b presents additional information involving another metallic component, which is generally rarely used in rendering the cerebral blood vessels. Professional radiographers semi-automatically eliminate this information during the final image acquisition for diagnosis based on 3D rendering of BS-CTA data. A method to eliminate the unnecessary information during 3D rendering of the brain vessel via continuous research and development is underway. Figure 8 illustrates the 3D rendering technique based on the results presented in Figure 7. Here, (a) represents a two-clip patient case and (b) a case of the coil. The proposed method improved the extraction of metallic components compared with conventional subtraction method. Here, the residual information in the metallic component region following the implementation of the proposed method was generated via reconstruction, artifact correction, and image registration, which may be used to reconstruct information of the metallic components during 3D rendering. However, it is irregular in each case and the metallic substances are completely removed as shown in the two-clip case (Figure 8). The viability of the proposed method was confirmed.

The overlap between the ground truth map (reference map) and the segmentation map of 40 patients using the proposed method yielded a mean DSC of 0.93 with an SD of ±0.05 (e.g., maximum value = 0.99, minimum value = 0.75, 95% CI = 0.91 to 0.95, and all *p* < 0.05), and the mean HD was 1.50 voxels with an SD of ±0.58 (e.g., maximum value = 5.95, minimum value = 0.12, 95% CI = 1.10 to 1.90, and all *p* < 0.05). Here, 28 patients with one clip, four patients with two clips, four patients with coils, and four patients with various metallic components were included as shown in Table 3.

**Figure 9:**
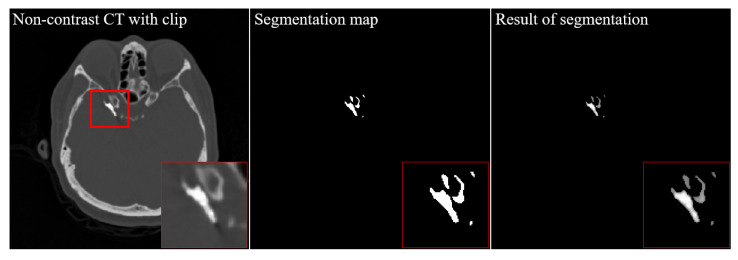
Segmentation of both metallic component and bone, which is very close to the target metal, using the proposed method.

Despite the outstanding results of the proposed method, the method has limitations, as shown in Figure 9. The proposed algorithm is based on the threshold method using the HU value and it is difficult to extract the perfect contour of the metal region. When the threshold value to segment the metal component is similar to the value of the neighborhood region (e.g., bone), this value may not be set low because it extracted not only vessel but also other components. In this respect, the results were unsatisfactory and this may require additional radiation exposure and greatly reduces the efficiency of diagnostic procedures. In addition, the insertion of metallic components such as clip, coil, and stent may result in metal artifacts, and insufficient results may be produced due to beam hardening phenomenon and scatter [26,27]. Since this can cause loss of information, we intend to improve the performance of the proposed method via ongoing research and development.

## 4. Conclusions

The work focuses on metallic component segmentation of cerebral CTA in order to provide meaningful information to improve the accuracy of diagnosis. The study indicates that a high-intensity index is required to set the ROI, which includes the metallic component and calculate the threshold level necessary to divide between the metallic component and the background regions such as the soft tissue, followed by 3D rendering of the vessel including the clip or coil. The clinical findings demonstrate that the proposed method resulted in effective segmentation and application in conventional BS-CTA systems. Nevertheless, accurate segmentation is limited by the presence of organs such as bones around a metallic substance, resulting in increased procedural time and unsatisfactory results. Recently, machine- and deep-learning approaches have gained attention in image restoration and segmentation, which are expected to improve the usefulness of the proposed method by overcoming challenges intrinsic to conventional BS-CTA. However, these approaches are often demanded on the huge dataset, and it takes a lot of time and effort to refine data for training. Therefore, we have a plan that gathers more patient data and build the training datasets, including the accurate binary maps for metal regions. Consequently, we intend to develop methods that can be applied in single-scan CT, such as DE-BS-CTA, instead of dual scanning protocols in order to reduce the patient levels of radiation exposure.

## Figures and Tables

**Table 1:** Scan parameters of cerebral non-contrast CT and contrast CTA.

Parameters	Dimensions
Tube potential (kV_p_)	100
Beam collimation (mm)	0.6
Beam pitch	0.8
Rotation time (s)	0.5
Scanning direction	Caudocranial
Slice thickness (mm)	0.75, 1
Matrix size	512×512×320
Section overlap (mm)	0.75, 1
Kernel	Hv 40, 49
Reconstruction	ADMIRE 1

**Table 2:** Dice similarity coefficient (DSC) and Hausdorff distance (HD) descriptive statistics are shown in Figure 5.

Parameters	Variables	
	DSC	HD
55th slice	0.88	1.35
56th slice	0.84	5.07
57th slice	0.90	4.33
58th slice	0.92	3.07
59th slice	0.98	1.02
60th slice	0.95	1.11
61st slice	0.85	2.75
62nd slice	0.94	2.54
63rd slice	0.98	1.92
Mean±SD	0.92±0.05	2.58±1.42
Median	0.92	2.54
(minimum, maximum)	(0.94, 0.98)	(1.02, 5.07)
95% CI	(0.88, 0.96)	(0.81, 3.00)

**Table 3:** Analysis of DSC and HD variables of 40 patients.

Parameters	Variables	
	DSC	HD
Mean±SD	0.93±0.05	1.50±0.58
Median	0.94	1.12
(minimum, maximum)	(0.75, 0.99)	(0.12, 5.95)
95% CI	(0.91, 0.95)	(1.10, 1.90)

## Data Availability

Not applicable.

## References

[1] Lell M.M., Anders K., Uder M., Klotz E., Ditt H., Vega-Higuera F., Boskamp T., Bautz W.A., Tomandl B.F. (2006). New techniques in CT angiography. Radiographics.

[2] Lu L., Zhang L.J., Poon C.S., Wu S.Y., Zhou C.S., Luo S., Wang M., Lu G.M. (2012). Digital subtraction CT angiography for detection of intracranial aneurysms: Comparison with three-dimensional digital subtraction angiography. Radiology.

[3] Chen W., Xing W., Peng Y., He Z., Wang C., Wang Q. (2013). Cerebral aneurysms: Accuracy of 320–detector row nonsubtracted and subtracted volumetric CT angiography for diagnosis. Radiology.

[4] Rinkel G.J., Van Gijn J., Wijdicks E.F. (1993). Subarachnoid hemorrhage without detectable aneurysm. A review of the causes. Stroke.

[5] Saitoh H., Hayakawa K., Nishimura K., Okuno Y., Teraura T., Yumitori K., Okumura A. (1995). Rerupture of cerebral aneurysms during angiography. AJNR Am. J. Neuroradiol..

[6] Gonner F., Lövblad K.-O., Heid O., Remonda L., Guzman R., Barth A., Schroth G. (2002). Magnetic resonance angiography with ultrashort echo times reduces the artefact of aneurysm clips. Neuroradiology.

[7] Olsrud J., Lätt J., Brockstedt S., Romner B., Björkman-Burtscher I. (2005). Magnetic resonance imaging artifacts caused by aneurysm clips and shunt valves: Dependence on field strength (1.5 and 3 T) and imaging parameters. J. Magn. Reson. Imaging.

[8] Hwang S.B., Kwak H.S., Han Y.M., Chung G.H. (2011). Detection of intracranial aneurysms using three-dimensional multidetector-row CT angiography: Is bone subtraction necessary?. Eur. J. Radiol..

[9] Aulbach P., Mucha D., Engellandt K., Hädrich K., Kuhn M., Von Kummer R. (2015). Diagnostic impact of bone-subtraction CT angiography for patients with acute subarachnoid hemorrhage. Am. J. Neuroradiol..

[10] Lell M., Ditt H., Panknin C., Sayre J., Ruehm S., Klotz E., Tomandl B., Villablanca J. (2007). Bone-Subtraction CT Angiography: Evaluation of Two Different Fully Automated Image-Registration Procedures for Interscan Motion Compensation. Am. J. Neuroradiol..

[11] Nagayama Y., Nakaura T., Tsuji A., Urata J., Furusawa M., Yuki H., Hirarta K., Oda S., Kidoh M., Utsunomiya D. (2017). Cerebral bone subtraction CT angiography using 80 kVp and sinogram-affirmed iterative reconstruction: Contrast medium and radiation dose reduction with improvement of image quality. Neuroradiology.

[12] Kang M.-J., Park C.M., Lee C.-H., Goo J.M., Lee H.J. (2010). Focal iodine defects on color-coded iodine perfusion maps of dual-energy pulmonary CT angiography images: A potential diagnostic pitfall. Am. J. Roentgenol..

[13] Dinkel J., Khalilzadeh O., Phan C.M., Goenka A.H., Yoo A.J., Hirsch J.A., Gupta R. (2014). Technical limitations of dual-energy CT in neuroradiology: 30-Month institutional experience and review of literature. J. NeuroInterv. Surg..

[14] Alvarez R.E., Macovski A. (1976). Energy-selective reconstructions in X-ray computerised tomography. Phys. Med. Biol..

[15] McCollough C.H., Leng S., Yu L., Fletcher J.G. (2015). Dual- and multi-energy CT: Principles, technical approaches, and clinical applications. Radiology.

[16] Willemink M.J., Persson M., Pourmorteza A., Pelc N.J., Fleischmann D. (2018). Photon-counting CT: Technical Principles and Clinical Prospects. Radiology.

[17] Watanabe Y., Kashiwagi N., Yamada N., Higashi M., Fukuda T., Morikawa S., Onishi Y., Iihara K., Miyamoto S., Naito H. (2008). Subtraction 3D CT angiography with the orbital synchronized helical scan technique for the evaluation of postoperative cerebral aneurysms treated with cobalt-alloy clips. Am. J. Neuroradiol..

[18] Hegde A.H., Chan L.L., Tan L., Illyyas M., Lim W.E.H. (2009). Dual energy CT and its use neuroangiography. Ann. Acad. Med. Singap..

[19] Lev M., Gonzalez R. (2002). CT Angiography and CT Perfusion Imaging.

[20] Naruto N., Itoh T., Noguchi K. (2017). Dual energy computed tomography for the head. Jpn. J. Radiol..

[21] Otsu N. (1979). A threshold selection method from gray-level histograms. IEEE Trans. Syst. Man Cybern..

[22] Shibata E., Takao H., Amemiya S., Ohtomo K. (2017). 3D-printed visceral aneurysm models based on CT data for simulations of endovascular embolization: Evaluation of size and shape accuracy. Am. J. Roentgenol..

[23] Carass A., Roy S., Gherman A., Reinhold J.C., Jesson A., Arbel T., Maier O., Handels H., Ghafoorian M., Platel B. (2020). Evaluating white matter lesion segmentations with refined sørensen-dice analysis. Sci. Rep..

[24] Taha A.A., Hanbury A. (2015). An efficient algorithm for calculating the exact hausdorff distance. IEEE Trans. Pattern Anal. Mach. Intell..

[25] Karimi D., Salcudean S.E. (2019). Reducing the hausdorff distance in medical image segmentation with convolutional neural networks. IEEE Trans. Med. Imaging.

[26] Meyer E., Maaß C., Baer M., Raupach R., Schmidt B., Kachelrieß M. Empirical scatter correction (esc): A new CT scatter correction method and its application to metal artifact reduction. Proceedings of the IEEE Nuclear Science Symposuim & Medical Imaging Conference.

[27] Pjontek R., Önenköprülü B., Scholz B., Kyriakou Y., Schubert G.A., Nikoubashman O., Othman A., Wiesmann M., Brockmann M.A. (2015). Metal artifact reduction for flat panel detector intravenous CT angiography in patients with intracranial metallic implants after endovascular and surgical treatment. J. NeuroInterv. Surg..

